# Bone Tissue Regeneration in the Oral and Maxillofacial Region: A Review on the Application of Stem Cells and New Strategies to Improve Vascularization

**DOI:** 10.1155/2019/6279721

**Published:** 2019-12-30

**Authors:** Vivian Wu, Marco N. Helder, Nathalie Bravenboer, Christiaan M. ten Bruggenkate, Jianfeng Jin, Jenneke Klein-Nulend, Engelbert A. J. M. Schulten

**Affiliations:** ^1^Department of Oral Cell Biology, Academic Centre for Dentistry Amsterdam (ACTA), University of Amsterdam and Vrije Universiteit Amsterdam, Amsterdam Movement Sciences, Amsterdam, Netherlands; ^2^Department of Oral and Maxillofacial Surgery/Oral Pathology, Amsterdam University Medical Centers and Academic Centre for Dentistry Amsterdam (ACTA), Vrije Universiteit Amsterdam, Amsterdam Movement Sciences, Amsterdam, Netherlands; ^3^Department of Clinical Chemistry, Amsterdam University Medical Centers, Vrije Universiteit Amsterdam, Amsterdam Movement Sciences, Amsterdam, Netherlands

## Abstract

Bone tissue engineering techniques are a promising alternative for the use of autologous bone grafts to reconstruct bone defects in the oral and maxillofacial region. However, for successful bone regeneration, adequate vascularization is a prerequisite. This review presents and discusses the application of stem cells and new strategies to improve vascularization, which may lead to feasible clinical applications. Multiple sources of stem cells have been investigated for bone tissue engineering. The stromal vascular fraction (SVF) of human adipose tissue is considered a promising single source for a heterogeneous population of essential cells with, amongst others, osteogenic and angiogenic potential. Enhanced vascularization of tissue-engineered grafts can be achieved by different mechanisms: vascular ingrowth directed from the surrounding host tissue to the implanted graft, vice versa, or concomitantly. Vascular ingrowth into the implanted graft can be enhanced by (i) optimizing the material properties of scaffolds and (ii) their bioactivation by incorporation of growth factors or cell seeding. Vascular ingrowth directed from the implanted graft towards the host tissue can be achieved by incorporating the graft with either (i) preformed microvascular networks or (ii) microvascular fragments (MF). The latter may have stimulating actions on both vascular ingrowth and outgrowth, since they contain angiogenic stem cells like SVF, as well as vascularized matrix fragments. Both adipose tissue-derived SVF and MF are cell sources with clinical feasibility due to their large quantities that can be harvested and applied in a one-step surgical procedure. During the past years, important advancements of stem cell application and vascularization in bone tissue regeneration have been made. The development of engineered *in vitro* 3D models mimicking the bone defect environment would facilitate new strategies in bone tissue engineering. Successful clinical application requires innovative future investigations enhancing vascularization.

## 1. Introduction

To rehabilitate patients with critical-sized bone defects, surgical reconstructions are required. A critical-sized defect will not heal spontaneously or regenerate more than 10% of the lost bone during patients' lifetime [1]. These bone defects may result from systemic or local causes. Systemic conditions include congenital abnormalities [2], general diseases [3], and medications [4], while local conditions comprise inflammation [5] or traumatic injuries, such as accidents [6] or dental and surgical treatments. Dental treatments, such as tooth extraction [7], and surgical treatments, such as surgical resection of benign or malignant neoplasms [8], may lead to substantial jaw bone defects.

Bone grafting procedures are carried out to reconstruct a bone defect [9]. In these surgical procedures, autografts are still considered the “gold standard” due to the essential combination of osteogenic, osteoinductive, and osteoconductive properties. However, autografts have some disadvantages, e.g., donor site morbidity and limited amount of graft tissue. In some cases, bone substitutes, such as allografts, xenografts, and alloplasts, are used as alternatives for autologous bone grafts, but these bone substitutes lack osteogenic, osteoinductive, and angiogenic potential [10].

Unfortunately, the ideal bone regeneration technique and material have not yet been developed. However, recent developments in tissue engineering have led to new and better treatment options called “cellular bone tissue engineering.” In this approach, a scaffold with mesenchymal stem cells (MSCs) and/or osteoprogenitor cells of an external source is implanted into the bone defect site. The *ex vivo* seeded cells on the scaffold play a key role and orchestrate the mechanism of bone formation at the target site. Multiple techniques have been investigated, applying a variety of stem cell sources and cell processing protocols [11]. Furthermore, different scaffold types are used for carrying the cells [12].

The rationale behind the application of MSCs and/or osteoprogenitor cells is their key role in bone formation. Natural bone formation in the pre- and postnatal development of the oral and maxillofacial area is performed intramembranously by recruiting mesenchymal bone marrow cells. These cells undergo osteoblastic differentiation and initiate new bone formation in the defect site. In other words, this method is aimed at inducing bone regeneration by mimicking biologic processes that occur during embryogenesis [13, 14].

The mechanism by which MSCs promote bone regeneration can be directed by engraftment of the transplanted cells into the newly regenerated tissue, differentiating into osteoblasts that eventually will secrete osteoid and initiate mineralization [15–17]. In addition, MSCs can enhance bone regeneration indirectly by a paracrine effect, i.e., secretion of cytokines and growth factors such as transforming necrosis factor-*α* (TNF-*α*), platelet-derived growth factor (PDGF), interleukin-1 (IL-1), and IL-6. These secreted factors may recruit resident MSCs to the regenerated site [18, 19].

In cellular bone tissue engineering, MSCs are applied using two different approaches. The first approach is to directly transplant MSCs and/or osteoprogenitor cells combined with a scaffold (external scaffold) into the bone-defected site, which is a kind of an *in situ* tissue engineering [20, 21]. Autogenous particulate cancellous bone and marrow are used as the source of osteoprogenitor cells and MSCs. In this approach, the scaffold functions as a framework [22]. The second approach is to transplant MSCs that are isolated (usually from the patient), expanded *ex vivo*, seeded on adequate three-dimensional (3D) scaffolds (internal scaffolds), and proliferated and/or predifferentiated in controlled culture conditions [23]. Such a scaffold acts as a carrier of the cells and temporary matrix while the cells produce the extracellular matrix (ECM) that is required for bone formation [24].

A major challenge in bone tissue engineering is the vascularization of the implanted graft. Graft survival requires rapid and sufficient vascularization. Since the amount of oxygen is limited to a diffusion distance of only ~150-200 *μ*m from a supply blood vessel, cells lying beyond this physiological border suffer from hypoxia [25]. Under this condition, MSCs fail to survive, because they are not able to adapt their glucose consumption and do not possess the necessary glycolytic reserves to maintain their metabolism for more than three days [26]. New insights underline the importance of both oxygen and nutrients required for energy-related cellular metabolism and in the end cell survival. Regenerating tissue over 200 *μ*m exceeds the capacity of nutrient supply and waste removal from the tissue and, therefore, requires an intimate supply of vascular networks [25]. Neovascularization along with efficient supply of blood is a prerequisite to this end.

The aim of this review is to present and discuss the advancement of stem cell application, vascularization, and bone regeneration in the oral and maxillofacial region, with emphasis on the human jaw. Moreover, we propose new strategies to improve the current techniques, which may lead to feasible clinical applications.

## 2. Sources of Stem Cells

**Figure 1:**
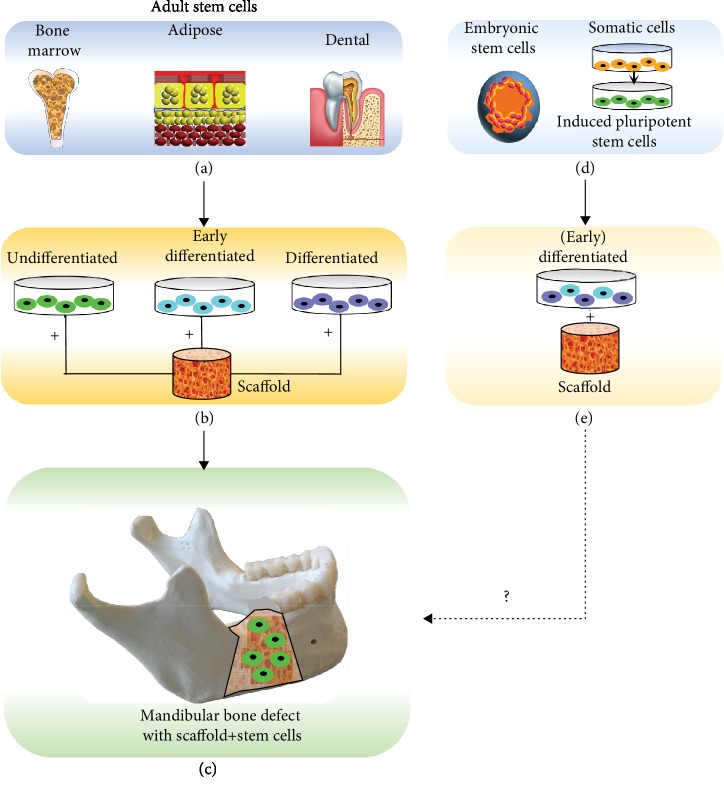
Overview of stem cell sources and their stage (undifferentiated, early differentiated, or differentiated) of application. Adult stem cells that are currently applied in clinical studies are retrieved from bone marrow, adipose, or dental tissue (a). These cells are applied in an undifferentiated, early differentiated, or differentiated stage seeded on a scaffold (b). The scaffold with the stem cells is applied in clinical trials to regenerate bone defects, such as mandibular bone defects (c). Embryonic stem cells and somatic stem cells, which are first stimulated into induced pluripotent stem cells (d), are applied in a (early) differentiated stage on a scaffold (e). Their application in clinical trials still needs to be envisioned (c). Note that in the mandibular bone defect shown (c), the stem cells are undifferentiated. However, the stem cells applied in such bone defects could be also early differentiated or differentiated.

Somatic stem cells, mainly mesenchymal stem cells (MSCs), that are applied in bone tissue engineering are isolated from various tissues. The clinically applied sources of stem cells in the oral and maxillofacial region originate from bone marrow, adipose tissue [27], and dental tissues [28, 29]. *In vitro* and *in vivo* animal studies reported on the application of embryonic stem cells (ESCs) [30–32] and induced pluripotent stem cells (IPSCs) [33] in bone tissue engineering. However, these ESCs and IPSCs raise several serious ethical and safety concerns, such as teratoma formation, which continue to impede clinical implementation [34]. In Figure 1, the different sources of stem cells and their different stages of application are illustrated: undifferentiated, early differentiated, or differentiated. The different stages of stem cells are categorized as follows:

- Undifferentiated: multipotent adult MSCs, pluripotent ESCs, or IPSCs
- Early differentiated: MSCs differentiated towards specific lineage, such as osteogenic lineage
- Differentiated: specialized cell, such as osteoblast

Clinically applicable tissue engineering involving stem cells is focused on the use of patient-derived (adult) stem cells that are undifferentiated, given that terminally differentiated cells are difficult to expand *ex vivo* relative to more highly proliferative stem/progenitor cells. The use of stem cells is also intended to achieve a complete physiological repair process that involves the MSC-mediated activation of not only bone formation but also neovascularization. Nevertheless, it is of pivotal importance to prohibit unwanted side effects such as teratoma formation which may occur by ESCs and IPSCs.

In the following, an overview of the currently *in vivo* applied stem cell sources is given. Besides, Table 1 provides an overview of the recent clinical trials, published between January 1, 2015, and November 1, 2019, with successful application of human-derived stem cells. “A successful application” was considered a significant outcome measurement due to the supplementation of MSCs specifically. The majority of these studies investigated bone formation as an outcome measurement based on radiography, (cone beam) computed tomography ((CB)CT), microcomputed tomography (micro-CT), or histomorphometric and/or histologic measurements. As a future direction, it would be interesting to investigate the vascularization in these cases, since enhanced vascularization would be expected in relation to the enhanced osteogenetic effects observed due to the supplementation of MSCs. A complete overview of all the clinical studies applying MSCs has been described earlier [35].

Bone marrow was the first source reported to contain MSCs [36]. Until today, adult bone marrow-derived stem cells (BMSCs) are the most frequently investigated type of MSCs in bone tissue engineering. Several successful applications of BMSCs in *vivo* have been reported in the oral and maxillofacial region (Table 1). There are two different interventions in the application of BMSCs: (1) the use of bone marrow aspirate (concentrated), a whole tissue fraction containing BMSCs, and (2) the use of *in vitro* cultivated BMSCs (expanded with or without differentiation factors) (Table 1). Concentrated bone marrow aspirate compared to noconcentrated aspirate seems to have a higher osteogenic potential *in vivo* [37]. An overview of the successful clinical trials performed with this cell source is shown in Table 1.

Several studies showed promising results applying BMSCs in surgical procedures in the oral and maxillofacial region. Some maxillary sinus floor elevation studies presented histomorphometrical data that showed increased new bone formation after 3 to 4 months compared to traditional methods using bone substitutes alone [38, 39]. Kaigler et al. showed accelerated bone regeneration in extraction sockets of teeth when applying BMSCs or gelatin sponge compared to the controls (saline-soaked gelatin sponge) [40]. Baba et al. conducted a phase I/II clinical trial involving ten patients with periodontitis, who required a surgical procedure for intrabony defects, applying bone marrow-derived stem cells with a biodegradable 3D-poly-lactic-acid-based scaffold and platelet-rich plasma. After 12 months, the bone defect showed clinically and radiographically significant improvement compared to conventional periodontal surgical procedures without application of stem cells. These results suggest successful clinical application in regenerating periodontal tissue, including bone tissue [41]. In alveolar cleft surgery, several clinical trials, mainly case reports, suggest promising results with the application of BMSCs, but complete reconstruction (bone fill) of extensive cleft defects has not been demonstrated [42, 43]. In contrast, Hermund et al. [44] showed no difference in bone density and height between a control group (graft composed of a mixture of bovine bone substitute and autologous bone particles) and a test group (same scaffold, supplemented with BMSCs that were retrieved from the tuberosity and cultivated *in vitro*) after maxillary sinus floor elevation.

Unfortunately, BMSC application comes with limitations: bone marrow aspiration is an invasive and painful procedure for the donor, and cell retrieval is scarce, since the frequency of BMSCs in human bone marrow is rather low (0.001%–0.01%) [45]. Consequently, fresh bone marrow aspirates may result in a too low number and concentration of BMSCs to exert substantial osteogenic effects [37]. Therefore, *in vitro* culture expansion is required to obtain sufficient numbers of cells for clinical application [46]. This cell expansion, however, needs to be done in a laborious, expensive, and time-consuming good manufacturing practice (GMP) laboratory. Other limitations comprise the loss of proliferative and differentiation capacities during cell expansion [47, 48] and an increased risk for pathogen contamination and genetic transformation [49, 50]. Last but not least, the number, proliferation, and differentiation potential of BMSCs decline with increasing age [51].

Adipose tissue-derived mesenchymal stem cells (ASCs) have opened appealing new possibilities in adult stem cell therapies. ASCs show many similarities with BMSCs with regard to surface marker profiles, multilineage potential, and growth properties [52]. However, in contrast to the other sources (bone marrow, dental, and embryonal), adipose tissue has the following advantages: (a) it has a high stem cell-to-volume ratio [53, 54], (b) the stem cell frequency is far less sensitive to ageing [55], (c) harvesting can easily be upscaled according to the need, and (d) it can be processed within a short time frame to obtain highly enriched ASC preparations (residing in the stromal vascular fraction [SVF]). Furthermore, the multipotent cells within the SVF attach very fast to the scaffold material, proliferate rapidly, and can be differentiated toward amongst others the osteogenic lineage [56, 57].

Helder and colleagues formulated the concept of the one-step surgical procedure (OSP) to apply ASCs in the regeneration of bone tissues [58]. After harvesting the adipose tissue by the surgeon, the SVF-containing ASCs can be seeded onto the scaffold material without culture expansion. Then the ASC scaffold construct can be implanted, all in the same surgical procedure. The obvious advantage of this one-step surgical procedure is not only its patient-friendliness but also its lower costs, since a second surgical intervention and expensive *in vitro* culturing steps can be avoided.

Multiple *in vitro* studies made important advancements in the application of ASCs in bone tissue engineering [32]. Recently, successful results were also obtained in clinical trials (Table 1). The results from a first clinical trial evaluating the application of ASCs showed that it is a feasible, safe, and effective treatment option in jaw bone regeneration [59]. Prins et al. showed in a split-mouth design that patients undergoing maxillary sinus floor elevation for dental implant placement benefitted from the application of ASCs. Bone and osteoid percentages were higher in study biopsies (SVF supplemented to different ceramic bone substitutes) than in control biopsies (ceramic only on contralateral side) (54). The additive effect of SVF supplementation was independent of the bone substitute *β*-tricalcium phosphate or biphasic calcium phosphate (hydroxyapatite/tricalcium phosphate) [59]. Khojasteh et al. [60] used ASCs derived from the buccal fat pad, *in vitro* cultivated, and seeded on demineralized bovine bone mineral (DBBM) and autologous bone (AB), in alveolar cleft reconstruction. Cone beam-computed tomography 6 months after the treatment showed more bone formation in the test group with supplementation of ASCs. Castillo-Cardiel et al. [61] treated mandibular condylar fractures with abdominal retrieved ASCs that were *in vitro* cultivated and injected at the fracture site. After 12 weeks of the surgical treatment, the test group with the supplemented ASCs had a 37% higher ossification rate compared to the traditional treatment (control group). A disadvantage of SVF harvesting so far is that it is performed under general anesthesia and requires (short) hospitalization. Also, postoperative care and complaints are to be regarded. However, clinical studies using local anesthesia are currently being undertaken, which may widen the applicability of this intraoperative approach.

Dental tissues provide several populations of stem cells, including the pulp of both exfoliated and adult teeth, periodontal ligament, and dental follicle [62]. Dental tissue-derived stem cells (DSCs) have generic mesenchymal stem cell-like properties such as self-renewal and multilineage differentiation into chondrogenic, osteogenic, and adipogenic cell lineages. In addition, DSCs also show neurogenic and angiogenic potential [62]. It has been demonstrated that DSCs have the ability to generate not only dental tissue such as dentine/pulp-like complexes but also bone tissue [63, 64]. Stem cells from human-exfoliated deciduous teeth exhibit higher proliferation rates and can be easier obtained compared to BMSCs [65].

However, published clinical studies with successful results are scarce (Table 1). D' Aquino et al. [66] used whole tissue fractions from periosteum tissue by mechanically disaggration, followed by soaking of a collagen sponge in the resulting disaggregated tissue. Calcification was enhanced in tooth socket preservation in the test group with DSCs supplemented to the collagen sponge, compared to the control group with unloaded collagen sponges. Monti et al. [67] used tissue fractions from the dental pulp, followed by soaking of a collagen sponge in a similar clinical model. Sixty days after grafting, the test site (supplemented with DSCs) showed stronger radiopacity when compared with the control site (collagen sponge). Histological analysis showed well-differentiated bone with Haversian system formation in the test site with more bone formation. Baena et al. [68] used whole tissue fractions from periosteum tissue seeded on a poly(lactic-co-glycolic acid) (PLGA) scaffold with hydroxyapatite (HA) in maxillary sinus floor elevation surgery. They showed an increased percentage of vital mineralized tissue in the group treated with both periosteum-derived stem cells and PGLA/HA, with respect to the control group of PGLA/HA or demineralized bovine bone mineral alone, as confirmed by histological analysis and radiographic evaluations at six months after the treatment. Ferrarotti et al. [69] showed clinical success after applying dental pulp stem cells on a collagen sponge in intrabony periodontal regeneration one year after treatment.

The question remains open whether in spite of the low numbers of cells, DSCs might become an attractive source of autologous SCs for bone regeneration. This source is being investigated with at least more than ten new trials underway (http://www.clinicaltrials.gov).

## 3. Vascularization in Bone Tissue Regeneration

Successful bone tissue regeneration requires rapid perfusion and integration of the implanted graft with the recipient vasculature. Neovascularization is achieved by both vasculogenesis and angiogenesis. Vasculogenesis is originally described as *de novo* blood vessel formation by differentiation and assembly of angioblastic progenitor cells during embryogenesis [70]. However, more recently, postnatal vasculogenesis is becoming evident as a major contributor to adult neovascularization. This type of postnatal vasculogenesis is defined as the incorporation of circulating endothelial progenitor cells (EPCs) into the microvascular endothelium of newly developing microvessels [71, 72].

EPCs are mainly located within the stem cell niche in bone marrow, along with some circulating populations in the peripheral blood. When injury or tissue damage occurs, EPCs are thought to mobilize from the bone marrow into the circulation and home to tissue repair sites under the guidance of signals such as hypoxia, growth factors, chemoattractant signals, and chemokines. EPCs then invade and migrate at the same sites and differentiate into mature endothelial cells (ECs) and/or regulate preexisting ECs via paracrine or juxtacrine signals [73].

Angiogenesis is defined as new blood vessel sprouting from preexisting vessels. The first step in this process is the activation of the host microvasculature at the implantation site by angiogenic growth factors, such as vascular endothelial growth factor (VEGF) or basic fibroblast growth factor [74]. These factors may originate from different sources. They may be produced by cells of the host tissue itself due to tissue injury during the implantation procedure or in consequence of an inflammatory response to the implanted graft.

The endothelial cells, which are lining blood vessels, allow the formation of new blood capillaries by the sprouting of an existing small vessel [75, 76]. Upon angiogenic activation, they start to produce matrix metalloproteinases, resulting in the degradation of their basement membrane [77]. This is the prerequisite for their subsequent migration into the surrounding interstitium, which is morphologically reflected by the formation of vascular buds and sprouts. The sprouts progressively grow into the implanted tissue construct and interconnect with each other to develop new blood-perfused microvascular networks [78]. The wall of these networks is finally stabilized by the production of extracellular matrix compounds and the recruitment of smooth muscle cells or pericytes [79].

Accordingly, successful vascularization of an implanted graft via vasculogenesis and angiogenesis is dependent on the coordinated sequence of various humoral and cellular mechanisms and, in particular, the close interaction between the host tissue and the implanted graft. This process allows tissue growth and repair by extending and remodeling the network of blood vessels [73, 80].

## 4. Vascularization Strategies in Bone Tissue Engineering

Several approaches to improve vascularization, through enhanced vasculogenesis and angiogenesis, of the implanted grafts are currently investigated. The classical vascularization strategies focus on the stimulation of vascular ingrowth into the implanted grafts from the surrounding host tissue by (i) optimizing the material properties of scaffolds and (ii) their bioactivation by incorporation of growth factor delivery systems or by cell seeding. However, endothelial cell migration and physiological growth of new blood vessels has been demonstrated not to be faster than ~5 *μ*m/h [81]. Therefore, these approaches face the problem that sufficient vascularization of the implanted graft requires a prolonged time period which is associated with major tissue loss due to hypoxic conditions.

**Figure 2:**
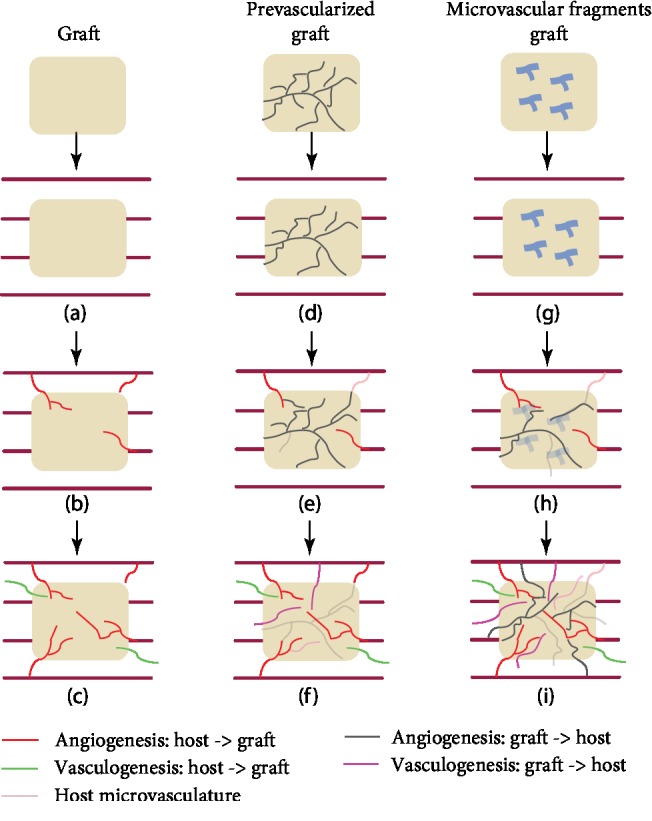
Overview of the three different vascularization strategies and their clinical results. First, a graft is implanted (a) which solely depends on the vascularization, angiogenesis, and vasculogenesis, from the host towards the graft (b). This results in insufficient vascularization of the graft (c). Second, a prevascularized graft is implanted in the host tissue (d). A high number of preformed microvessels have a suboptimal lifespan (e), resulting in less microvessels for vascularization from the graft towards the host (f). Third, microvascular fragments in the graft (g) develop rapidly into microvessels when implanted in the host tissue (h). They contribute to vascularization (angiogenesis and vasculogenesis) from the graft towards the host, which results in enhanced vascularization. Vascularization starts from two directions, i.e., from the graft and from the host tissue (i).

To overcome this problem, vascular ingrowth directed from the implanted graft towards the host tissue has been proposed to complement vascular ingrowth from the host tissue into the implanted graft. This can be achieved by incorporating the graft with either (i) preformed microvascular networks which can directly be perfused with blood by developing interconnections (inosculation) to the host microvasculature or (ii) microvascular fragments which rapidly develops into microvascular networks after transfer into the host tissue (Figure 2). In the following, an overview of the current possibilities and future perspectives on the above-mentioned strategies to enhance vascularization in bone tissue engineering is provided.

### 4.1. Material Properties of Scaffolds

The characteristics of the scaffold material play an important role in angiogenesis of the graft. Many different scaffold materials for bone tissue engineering have been investigated *in vivo* and *in vitro*, e.g., polymers, bioactive ceramics, and hybrids (composites) [12].

The chemical composition of scaffold materials has been shown to influence the angiogenic process at the implantation site. For instance, poly(lactic-co-glycolic acid) (PLGA), hydroxyapatite (HA), and dentin scaffolds show a slight inflammatory response after implantation, inducing marked angiogenic response and a good vascularization of the grafts after 14 days [78, 82]. In contrast, collagen-chitosan-hydroxyapatite hydrogel scaffolds of identical architecture induce severe inflammation, resulting in apoptotic cell death within the surrounding tissue and a complete lack of ingrowth of newly formed microvessels [78]. Polyurethane scaffolds, which exhibit an excellent *in vivo* biocompatibility, have been shown to be characterized by a poor vascularization [83]. These findings indicate that scaffold materials with slightly proinflammatory properties may stimulate the angiogenic host tissue response to the implanted scaffold material.

Combinations of biomaterials have been investigated to improve the scaffold properties. Composites consist of a combination of two or more materials with different properties, each displaying only some advantages and specific drawbacks. Polymer-ceramic composites have been successful in bone regeneration, exceeding the results obtained when these materials are used separately, showing improved mechanical and biological results [84]. The combination of PLGA (combination of poly lactide and polyglycolide) and HA or *β*-TCP allows to overcome the problems due to PLGA's acidic degradation products that may induce tissue necrosis and negatively affect neoangiogenesis, since HA and *β*-TCP neutralize the acidic degradation products of PLGA [85].

Not only the chemical composition but also the architecture of scaffolds is an important determinant for adequate vascularization [86]. It should contain distributed, interconnected pores and display a high porosity in order to ensure cell penetration, vascular ingrowth, nutrient diffusion, and waste product elimination [87]. Another key component to allow proper cell colonization (cells bound to ligands within the scaffold) is the mean pore size [88]. The minimum recommended pore size for a scaffold is 100 *μ*m [89] based on the early work of Hulbert et al. [90], but subsequent studies have shown better osteogenesis for implants with pores > 300 *μ*m [91, 92]. Relatively larger pores favor direct osteogenesis, since they allow vascularization and high oxygenation, while smaller pores result in endochondral ossification, although the type of bone ingrowth depends on the biomaterial and the geometry of the pores. There is, however, an upper limit in porosity and pore size set by constraints associated with mechanical properties [86, 93].

### 4.2. Bioactivation of the Scaffold by Incorporation of Growth Factors or Cell Seeding

A common strategy to improve scaffold vascularization is the stimulation of the angiogenic host tissue response at the implantation site by incorporation of angiogenic growth factors. For this purpose, VEGF [94, 95], basic fibroblast growth factor [96], platelet-derived growth factor [97], and angiogenin [98] are the most frequently used factors. However, there are continuing concerns about the cost of multiple cytokines and delivery, potential toxicity, and suboptimal endothelial migration in large tissue grafts.

Another important aspect to consider is that many angiogenic growth factors are known to be released spontaneously by cells under stress-related conditions, including hypoxia. Due to hypoxia, bone-derived osteoblast-like cells as well as bone marrow mesenchymal stem cells (BMSCs) are known to liberate growth factors such as VEGF. Based on this cellular mechanism, an accelerated vascularization of scaffolds is also achieved by seeding the scaffolds with differentiated tissue-specific cells [99, 100] or multipotent stem cells [101, 102]. Although BMSCs are known to have the potential to differentiate into defined vascular cells, it has been shown that the observed acceleration of vascularization at 14 days *in vivo* more strongly depends on the liberation of VEGF by the seeded cells than the differentiation potential of the BMSCs [99]. Even though there is significant acceleration of vascularization after cell seeding, Tavassol et al. [100] showed that the majority of seeded osteoblast-like cells died within the observation period of 14 days after *in vivo* implantation of PGLA scaffolds seeded with osteoblast-like cells. This indicated that this method alone is not sufficient to accelerate the vascularization to ensure the survival of seeded cells. Qu et al. [103] showed that genetically modified cells could have a long-term expression of angiogenic growth factors, independently from their state of hypoxia. They transfected BMSCs with basic fibroblast growth factor seeded on a composite scaffold in a calvarial critical-sized defect model in rats. It accelerated vascularization and bone regeneration at 4 and 8 weeks compared with the controls. However, it was also suggested that overexpression of angiogenic growth factor VEGF may cause a global reduction in bone quantity, consisting of thin trabeculae of immature matrices [104].

### 4.3. Preformed Microvascular Networks

Different approaches to prevascularize the graft *in vitro* by seeding of vessel-forming cells onto scaffolds are being investigated. After seeding onto the scaffold, these cells rapidly assemble into immature microvessels. In contrast to the above-mentioned approaches that focus on the stimulation of vascular ingrowth into the implanted graft, prevascularization is aimed at generating preformed microvascular networks inside the graft prior to their implantation. After implantation, these networks can be rapidly perfused with blood by inosculation with the surrounding host microvasculature [80].

Proangiogenic cells, such as endothelial cells, endothelial progenitor cells, and mural cells (pericytes and smooth muscle cells), are widely used as cell source. Other cell sources including adult stem cells, such as pluripotent mesenchymal stem cells from bone marrow [105, 106] or adipose tissue [106–108], and induced pluripotent stem cells [109] are also suggested as suitable sources for this purpose.

Originally, endothelial and endothelial progenitor cells were used for the formation of blood vessels, but this resulted in blood vessels with suboptimal lifespan [110]. Due to a limited number of transplanted vascular cells surviving for a prolonged duration, neovasculature fails to recruit the obligatory perivascular cells including mural cells and consequently does not resemble native, multilayered mature microvessels [111]. To overcome this problem, gene transfection to improve the survival and proliferation of the used vascular cells has been suggested [110, 112]. However, this genetic manipulation bears an oncogenic risk [113].

A better alternative being investigated seems to be the cocultivation of endothelial cells with mural cells. These cells are crucial for the stabilization, maturation, and long-term survival of newly formed microvessels. Koike et al. [114] demonstrated stable microvascular networks, which survived for one year *in vivo*, through cocultivation of human umbilical vein endothelial cells (HUVECs) with mural precursor cells. This is in contrast to microvessels engineered with HUVECs alone, which rapidly regressed after 60 days [110]. However, limitations of cell-based prevascularization approaches are that these approaches usually need complex and time-consuming cell isolation and cultivation procedures. Besides, their safety and success are highly sensitive to the quality of the cell isolates, the applied seeding strategy, and the number of cells seeded. Multiple studies reported on a critical optimum ratio between vascular cells and tissue-specific cells within a construct [115, 116]. Therefore, their clinical application is difficult to envision.

### 4.4. Microvascular Fragments (MF)

Prevascularization methods by cell seeding using cellular isolates may result in uncertain outcomes. Moreover, the correct ratio of cells to be used is difficult to determine. This led to a novel concept exploiting the use of microvascular fragments (MF) isolated from adipose tissue by short (5-10 min) digestion [117–119]. MF is a mixture of arteriolar, capillary, and venular vessel segments [120]. Several studies successfully isolated MF from mice [117, 118] and human [119] and transplanted adipose tissue-derived MF in animals. These studies further demonstrated that these fragments rapidly develop stable, blood-perfused microvascular networks after implantation into the host tissue. In culture, MF have been shown to release the proangiogenic factors vascular endothelial growth factor (VEGF) and basic fibroblast growth factor (bFGF) [121, 122]. In addition, microvascular fragments contain stem cell antigen (Sca)-1/VEGFR-2-positive endothelial progenitor cells and mesenchymal stem cells expressing common markers, such as CD44, CD73, CD90, and CD117 [123]. It has been speculated that the high vascularization potential of microvascular fragments is (partly) caused by these stem cell populations. Compared to the above described cell seeding strategies to generate *in vitro* preformed microvascular networks, the enzymatic digestion period for the isolation of microvascular fragments is much shorter (5-10 min) than that of single source cells and does not require complex and time-consuming *in vitro* incubation periods. Moreover, MF can also be obtained from patients in a one-step surgical procedure with a liposuction technique under local anesthesia [124].

However, the MF procurement does not avoid the regulatory burden of using stem cell preparations obtained by enzymatic digestion, which are considered “more than minimally manipulated” by the FDA and the European counterpart the EMA. Therefore, recently, much effort was put in the development of mechanical disruption of the tissue creating microfragmented adipose tissue/nanofat (MFAT/NFAT) (reviewed in Trivisonno et al.'s study [125]).

Strikingly, it was found that the microfragmentation of the adipose tissue, which kept the microarchitecture (extracellular matrix with embedded mesenchymal stem cells and microvascular fragments) of the fat intact but disrupts most mature adipocytes, showed a remarkable enrichment of blood vessel-stabilizing pericytes and release of many more growth factors and cytokines involved in tissue repair and regeneration, noticeably via angiogenesis, compared to enzymatically obtained SVF [126]. Moreover, the microfragmented adipose tissue maintained strong angiogenic and anti-inflammatory properties [127]. Autologous transplantation of such mechanically processed adipose tissue has been used with success in multiple indications, spanning a.o. cosmetics [128, 129], orthopedics [130, 131], and proctology [132].

## 5. Future Directions

Future investigations in cellular bone tissue engineering applications should be focused on enhancing vascularization, since adequate vascularization is a prerequisite for successful clinical bone regeneration. Moreover, due to existing discrepancies in the way human MSC are harvested and whether they are either directly applied without cultivation or isolated and cultured *ex vivo*, in addition to donor-dependent variability regarding the bone forming potency, further investigations are needed to standardize the production and quality of stem cells for therapeutic applications.

A promising future direction for cellular tissue engineering in jaw bone reconstruction with feasible clinical application is the use of the stromal vascular fraction (SVF) of human adipose tissue. SVF is considered a “single source” for cellular tissue engineering due to its heterogeneous population of essential cells, i.e., multipotent stem cells and progenitor cells, including endothelial cells, stroma cells, pericytes, preadipocytes, and hematopoietic cells. SVF also contains macrophages, which secrete a multitude of vascular growth factors and cytokines [133].

The adipose stem cells (ASCs) in SVF have been shown to attach, proliferate, and osteogenically differentiate on calcium phosphate scaffolds [134] and secrete a multitude of growth factors [57]. ASCs not only have been shown to have osteogenic potential *in vivo* [59] but also demonstrated angiogenic potential crucial for bone tissue engineering applications in mice [135]. This is supported by *in vitro* observations that ASCs in SVF secrete a variety of angiogenic and antiapoptotic growth factors [136] and that SVF is highly enriched with CD34+CD45−cells. The CD34+ cells are capable of stimulating angiogenesis and are involved in neovascularization processes that facilitate healing of ischemic tissues in mouse models [137]. Moreover, it has been demonstrated that if cultured within 3D scaffolds, the combination of endothelial cells and stromal cells derived from the SVF assembles into vascular structures, thus actively contributing to the vascularization of tissue-engineered bone grafts and stimulating their engraftment *in vivo* [124].

**Figure 3:**
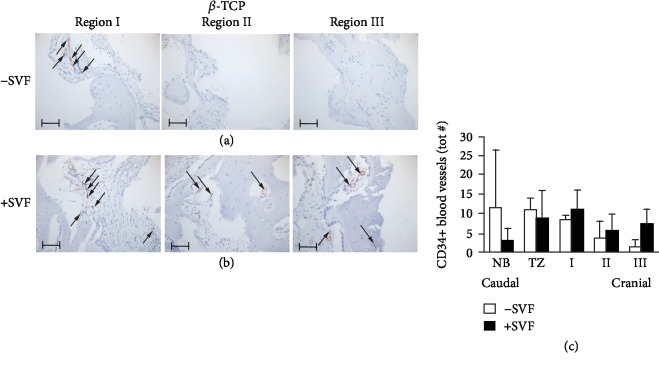
Immunohistochemical analysis of CD34, a marker of endothelial cells as well as stem cells such as endothelial progenitor stem cells and hematopoietic stem cells, of a maxillary bone biopsy from a patient treated with *β*-TCP (a–c). Magnification: 200x. The scale bar represents 100 *μ*m. The total number of CD34+ blood vessels of selected bone biopsies taken from control sides without stem cells (white bars; *n* = 3) and study sides with stem cells (black bars; *n* = 4) from patients treated with *β*-TCP (b). *β*-TCP: *β*-tricalcium phosphate; NB: native bone; TZ: transition zone; SVF: stromal vascular fraction; tot #: total number (adapted from Farré-Guasch E, Bravenboer N, Helder MN, Schulten EAJM, Ten Bruggenkate CM, Klein-Nulend J, 2018, Materials, 11, 161).

A first clinical trial confirmed that SVF/ASCs are capable to enhance bone and blood vessel formation [59, 138]. The study group (bone substitute [calcium phosphate] combined with SVF/ASCs) showed a higher bone mass that positively correlated with blood vessel formation versus the control group (only bone substitute) in a maxillary sinus floor elevation model [138]. Immunohistochemical analysis of CD34, a marker of endothelial cells as well as stem cells such as endothelial progenitor stem cells and hematopoietic stem cells, revealed a higher number of CD34+ blood vessels in the SVF-supplemented group (SVF+) than the bone substitute-only group (SVF-) (Figure 3), indicating a proangiogenic effect of the SVF. In addition, the vasculogenic effect of the SVF has been indicated *in vitro* [139].

Further investigations should also address the possibilities to enhance the osteogenic capacity of the ASCs within the treatment time of the “one-step surgery.” *In vitro* results of short (minutes) incubation of ASCs with a low dose of bone morphogenetic protein-2 (BMP-2) before seeding the cells on the scaffold (*β*-TCP and BCP) showed promising results; i.e., proliferation and osteogenic differentiation were enhanced by BMP-2 pretreatment, with concomitant downregulation of adipogenic gene expression. Stimulated gene expression of the osteogenic markers core binding factor alpha 1, collagen-1, osteonectin, and osteocalcin in the seeded ASCs was observed [134].

Recently, several studies suggested that adipose tissue-derived microvascular fragments (MF) show higher vascularization potential than SVF [118, 126]. However, further *in vitro* and *in vivo* research needs to confirm these findings. The MF and MFAT/NFAT variants of adipose tissue may spur future developments in particular for homologous applications since the regulatory burden can be avoided and the angiogenic, anti-inflammatory, and regenerative growth factor secretion properties appear at least equal but likely even higher than collagenase-digested SVF [126, 127].

The major clinical benefit of applying adipose tissue-derived SVF, MF, or MFAT/NFAT compared to other single-cell sources is that a native mixture of essential cells can be harvested in large quantities in a one-step surgical procedure. This makes clinical application of adipose tissue-derived SVF or MF feasible, due to its lower morbidity rate and shorter treatment duration compared to the traditional treatment options, such as autologous bone harvesting, bone marrow-derived stem cells, and endothelial cells.

Appropriate *in vitro* 3D models of bone defects to investigate cellular bone tissue engineering techniques, and specifically vascularization, are lacking. Such models would enhance the understanding of the interaction of cells with the host environment for osteogenesis and angiogenesis. Moreover, it would facilitate new possibilities for vascularization strategies. Currently exploited 2D-models and *in vivo* animal models have several limitations, including controllability, reproducibility, and flexibility of design. Recently, novel strategies in 3D-models are investigated to mimic human physiology *in vitro*, including bone niche-on-a-chip and bone bioreactors [140].

## 6. Conclusions

Important advancements have been made regarding the application of stem cells and the development of new strategies to improve vascularization in bone tissue engineering. However, adequate graft vascularization, which is a prerequisite to successful bone regeneration, is still considered a major challenge. The use of SVF of human adipose tissue seems to be a promising source for bone tissue engineering due to its heterogeneous population of essential cells for osteogenesis and angiogenesis. Besides, adipose tissue-derived MF is suggested as a promising cell source, due to its correct native cell ratios, for vascularization strategies. SVF, MF, and MFAT/NFAT are treatment options with clinical feasibility due to their large quantities that can be harvested and applied in a one-step surgical procedure. Appropriate *in vitro* models to study bone tissue engineering are lacking. Engineered *in vitro* 3D models mimicking the bone defect environment are crucial to facilitate new bone regeneration strategies. Successful bone reconstruction in the oral and maxillofacial region, using bone tissue engineering techniques, requires innovative future investigations focusing on the enhancement of vascularization.

## Figures and Tables

**Table 1:** Overview of clinical trials applying human-derived stem cells for bone tissue engineering applications/investigations to demonstrate *in vivo* possibilities.

Stem cell source	Intervention	Scaffold material	Clinical procedure	Reference
Bone marrow				
Posterior iliac crest	Aspirate concentrated	FDBA, PRP	Maxillary sinus floor elevation	Bertolai et al. [141]
Posterior iliac crest	Aspirate concentrated	DBBM	Maxillary sinus floor elevation	Pasquali et al. [142]
Posterior iliac crest	*In vitro* cultivation	*β*-TCP	Maxillary sinus floor elevation	Kaigler et al. [39]
Posterior iliac crest	*In vitro* cultivation	*β*-TCP	Alveolar cleft reconstruction	Bajestan et al. [43]
Posterior iliac crest	Aspirate concentrated	COL, PRF, nano-HA	Alveolar cleft reconstruction	Al-Ahmady et al. [143]
Posterior iliac crest	*In vitro* cultivation	HA-SI	Alveolar cleft reconstruction	Khalifa and Gomaa [144]
Posterior iliac crest	Aspirate concentrated	COL, CGF	Jaw defect reconstruction (after enucleation of cyst)	Talaat et al. [145]
Tuberosity	*In vitro* cultivation	PLA, PRP	Periodontal intrabony defect regeneration	Baba et al. [41]

Adipose tissue				
Abdominal	Aspirate concentrated into SVF	*β*-TCP or BCP	Maxillary sinus floor elevation	Prins et al. [59]
Buccal fat pad	*In vitro* cultivation	DBBM, AB	Alveolar cleft reconstruction	Khojasteh et al. [60]
Abdominal	*In vitro* cultivation	—	Mandibular condylar fracture regeneration	Castillo-Cardiel et al. [61]

Dental tissue				
Periosteum	Mechanical disaggregation of sample tissue	PLGA, HA	Maxillary sinus floor elevation	Baena et al. [68]
Pulp	Mechanical disaggregation of sample tissue	COL	Tooth socket preservation	Monti et al. [67]
Periosteum	Mechanical disaggregation of sample tissue	COL	Tooth socket preservation	D' Aquino et al. [66]
Pulp	Mechanical disaggregation of sample tissue	COL	Intrabony periodontal defects	Ferrarotti et al. [69]

PLA: polylactide acid; *β*-TCP: beta-tricalcium phosphate; FDBA: freeze-dried bone allografts; PRP: platelet-rich plasma; BCP: biphasic calcium phosphate (hydroxyapatite/tricalcium phosphate); DBM: demineralized bone matrix; AB: autologous bone; COL: collagen sponge; CGF: concentrated growth factor; HA: hydroxyapatite; PLGA: poly(lactic-co-glycolic acid); SI: silica.
